# Excess cerebellar granule neurons induced by the absence of p75NTR during development elicit social behavior deficits in mice

**DOI:** 10.3389/fnmol.2023.1147597

**Published:** 2023-05-25

**Authors:** Juan Pablo Zanin, Mansi A. Pandya, Diego Espinoza, Wilma J. Friedman, Michael W. Shiflett

**Affiliations:** ^1^Department of Biological Sciences, Rutgers, The State University of New Jersey, Newark, NJ, United States; ^2^Department of Psychology, Rutgers, The State University of New Jersey, Newark, NJ, United States

**Keywords:** p75NTR, granule cell precursor (GCPs), neurotrophins, cerebellum, social behavior

## Abstract

**Introduction:**

Recently, the cerebellum has been implicated with non-motor functions, including cognitive and emotional behavior. Anatomical and functional studies demonstrate bidirectional cerebellar connections with brain regions involved in social cognition. Cerebellar developmental abnormalities and injury are often associated with several psychiatric and mental disorders including autism spectrum disorders and anxiety. The cerebellar granule neurons (CGN) are essential for cerebellar function since they provide sensorimotor, proprioceptive, and contextual information to Purkinje cells to modify behavior in different contexts. Therefore, alterations to the CGN population are likely to compromise cerebellar processing and function. Previously we demonstrated that the p75 neurotrophin receptor (p75NTR) was fundamental for the development of the CGN. In the absence of p75NTR, we observed increased proliferation of the granule cell precursors (GCPs), followed by increased GCP migration toward the internal granule layer. The excess granule cells were incorporated into the cerebellar network, inducing alterations in cerebellar circuit processing.

**Methods:**

In the present study, we used two conditional mouse lines to specifically delete the expression of p75NTR in CGN. In both mouse lines, deletion of the target gene was under the control of the transcription factor Atoh-1 promotor, however, one of the lines was also tamoxifen-inducible.

**Results:**

We observed a loss of p75NTR expression from the GCPs in all cerebellar lobes. Compared to control animals, both mouse lines exhibited a reduced preference for social interactions when presented with a choice to interact with a mouse or an object. Open-field locomotor behavior and operant reward learning were unaffected in both lines. Lack of preference for social novelty and increased anxiety-related behavior was present in mice with constitutive p75NTR deletion; however, these effects were not present in the tamoxifen-inducible mice with p75NTR deletion that more specifically targeted the GCPs.

**Discussion:**

Our findings demonstrate that alterations to CGN development by loss of p75NTR alter social behavior, and contribute to the increasing evidence that the cerebellum plays a role in non-motor-related behaviors, including social behavior.

## Introduction

Traditionally, the cerebellum has been associated with motor balance and coordination, yet it is also involved in cognitive and emotional behavior. *In vivo* experimental data demonstrated the importance of the cerebellum in non-motor responses such as reward expectation (Wagner et al., 2017), decision-making (Blackwood et al., 2004; Deverett et al., 2018), and social interaction (Van Overwalle et al., 2014, 2020; Carta et al., 2019). Functional MRI in humans demonstrated that activation of the cerebellum is associated with addiction (Miquel et al., 2009; Moulton et al., 2014) and social cognition (Van Overwalle et al., 2014). Consistent with these findings, cerebellar developmental abnormalities and injury are often associated with several psychiatric and mental disorders including autism spectrum disorders (ASD) (Bauman and Kemper, 1985; Bailey et al., 1998; Fatemi et al., 2002; Whitney et al., 2008; Limperopoulos et al., 2009; Wang et al., 2014), schizophrenia (Andreasen and Pierson, 2008; Yeganeh-Doost, 2011; Parker et al., 2014), and anxiety (Hilber et al., 2004; Phillips, 2015; Moreno-Rius, 2018).

The cerebellum is well placed to influence non-motor behavior since it receives and sends information to many non-motor areas in the brain. For instance, a monosynaptic pathway between the cerebellum and the ventral tegmental area (VTA) was recently identified, suggesting the cerebellum can modulate reward circuitry with implications for social behavior (Carta et al., 2019). Sensory and motor information flow through the cerebellum via two pathways: (1) contextual sensory/motor information is provided to the CGN by the mossy fibers (MF); and (2) event inputs coming from the inferior olive transit via a single climbing fiber (CF). Both pathways converge in the Purkinje cells (PC), and this is likely one of the main reasons why this is the most studied cell population in the cerebellum; however, less attention has been paid to the other cell populations that feed information to PC, such as the CGN population.

The neurotrophin receptor p75 (p75NTR) is expressed throughout development as early as the blastocyst stage and continues to be expressed throughout development in different cell populations (Moscatelli et al., 2009; Vandamme and Berx, 2019). The function in each cell population depends on the cellular context, including the nature of the ligand, the receptor complex formed, and the intracellular pathway involved. p75NTR is abundantly expressed in the granule cell precursors (GCPs) in the developing cerebellum. Previously, we demonstrated that the absence of p75NTR specifically from cerebellar GCPs was sufficient to accelerate the cell cycle of the GCP, increasing the level of proliferation in this cell population compared to WT mice (Zanin et al., 2016, 2019). Moreover, the absence of p75NTR was also sufficient to allow an increase in GCP migration to the internal granule layer (IGL) (Zanin and Friedman, 2022). The excess neurons are incorporated into the cerebellar circuitry, affecting cerebellar network activity, characterized by the increased firing activity of the Purkinje cells (Zanin et al., 2019). These mice showed deficits in eyeblink conditioning, an associative learning task highly dependent on the cerebellum (Zanin et al., 2019). P75NTR is expressed in the proliferating GCPs in the external granule layer (EGL) of every lobe during cerebellar development, and, similarly, the increased proliferation mentioned above is observed in the entire cerebellum. Since the cerebellum has recently been associated with cognitive and emotional behavior, the cerebellar defects observed in the p75NTR conditional KO mouse suggest that other behaviors might be compromised in these animals. In the present work, using two different p75NTR conditional mouse lines, we evaluated the behavioral consequences of the specific deletion of p75NTR from the GCPs. Our analysis suggests that the deletion of p75NTR from GCPs during cerebellar development induces alterations in the social behavior of these animals.

## Materials and methods

### Subjects

Three- to four-month-old mice, both male and female, were used in all experiments. No sex difference was observed for any of the tests used. The same animals were used for the different behavioral tests, except the instrumental learning tasks, which were run on a separate cohort of animals. The order of the tests was as follows: 1. Open Field; 2. Elevated Zero Maze; 3. Light Avoidance Test; 4. Grooming Behavior; 5. Novel Object Recognition; 6. Social Novelty Test.

### Conditional deletion of p75NTR

To remove the expression of p75NTR from GCPs in the cerebellum, we mated floxed p75NTR mice (*p75*^FL/FL^*)* (Bogenmann et al., 2011) with *Atoh1^Cre^* mice (Jackson Labs. B6.Cg-Tg(P75Atoh1-Cre)1Bfri/J. RRID:IMSR_JAX:011104) (Matei et al., 2005). The genotype of each mouse was confirmed by PCR. The absence of p75NTR specifically from the EGL was confirmed by immunohistochemistry. For all behavioral analysis we used littermate mice, control mice were p75 with both floxed alleles (p75FL), conditional KO mice were also p75FL/FL with the expression of the Cre enzyme under the control of the Atoh1 promotor (p75Atoh1-Cre).

### Tamoxifen inducible p75NTR deletion

To remove expression of p75NTR from GCPs in the cerebellum at specific developmental stages, we mated floxed p75NTR mice (*p75*^FL/FL^*)* (Bogenmann et al., 2011) with p75Atoh1-CreERTM mice (RRID:MMRRC_029581-UNC) (Chow et al., 2006). The *p75Atoh1-CreERTM* generated was crossed with a Td-Tomato reporter mouse [Jackson Lab Gt(ROSA)26Sortm9(CAG-tdTomato)Hze; RRID:IMSR_JAX:007905] (Song et al., 2010) to allow assessment of the recombination efficiency following tamoxifen treatment. Tamoxifen was delivered via oral gavage to pregnant females at E17/18. Each female received one dose of tamoxifen (300 mg/kg of tamoxifen). Tamoxifen was dissolved in Corn oil (Sigma Cat # C8267). For all behavioral analysis we used mice with the same genotype (p75Atoh1-CreERTM) with or without tamoxifen exposure.

### Open field assessment

The open field apparatus (Stoelting Co., Wood Dale, IL, USA) is 40 cm x 40 cm (w/d) and constructed of gray plastic walls and a gray metal base. Mice were placed in the center of the open field and removed after 30 min. A video camera mounted above the open field captured video footage, which was processed to extract movement data using Noldus Ethovision XT v.11.5 (Noldus Information Technology, Leesburg VA, USA).

### Elevated zero maze

An elevated zero maze (Stoelting) measuring 50 cm in diameter and raised 50 cm from the floor was used. Mice were placed in one of the closed arms of the maze and removed after 5 min. A video camera mounted above the maze recorded mouse movement. Analysis of mouse movement in the elevated zero maze and time spent in the open arms of the arena was carried out using Noldus Ethovision v 11.5.

### Light-dark transition test

Mice were placed in a two-chambered arena (Med Associates, Fairfax, VT, USA). Each chamber measured 20.46 × 16.5 × 21.3 cm (L × W × H) with clear plastic walls and a stainless-steel floor. An automatic door separated each chamber. One chamber was covered with black material and was designated the dark chamber. The light chamber had a light source over the arena, which was lit to approximately 200 lumens on the floor. Mice were placed in the dark chamber for 2 min before the door was opened. The latency to enter the light chamber, and the amount of time spent in that chamber were recorded with a photobeam array installed in the chamber.

### Self-grooming assessment

Mice were placed in a standard polycarbonate mouse container identical to their home cage. The container was filled with approximately 1 cm of wood chip bedding. A video camera recorded mouse activity. Two observers blind to the subject genotype viewed mouse videos and scored them for self-grooming behavior using Noldus Ethovision 11.5.

### Novel object recognition test

Novel object recognition testing was based on previously described procedures (Shiflett, 2017). Mice were tested in an open field arena, as described above. During the sample phase, two identical objects (plastic bath toys) were placed in opposite corners of the arena 10 cm from the nearest walls. Mice were placed in the center of the arena and allowed to freely investigate both objects for 10 min after which they were returned to their home cage for 60 min. During the 5-min test phase, mice encountered one “familiar” object from the sample phase and a novel object. The number of sniffs on the familiar and novel objects was quantified from video footage.

### Social novelty test

Preference for social novelty was tested in a three-chambered arena, modified from that previously described (Shiflett, 2017). Each of the three chambers of the arena was equally sized and separated from each other by a plexiglass barrier. A small hole allowed passage between the chambers. Mice were first habituated to the empty arena for 30 min. During the 10-min sample phase, an unfamiliar male mouse was confined to one of the chambers by a small wire cage placed over it and the test mouse was allowed to freely roam the apparatus for 10 min. The opposite chamber contained a wire cage with no mouse. During the test phase, the mouse from the sample phase was returned to the apparatus and confined to one chamber. This mouse was designated the “familiar” mouse. An unfamiliar mouse was confined to the opposite chamber. The chamber where the familiar and novel mice were localized was randomized across subjects. The test mouse was placed in the center of the arena and allowed to freely roam the apparatus for 10 min. The amount of time spent in each chamber was quantified using Noldus Ethovision. The number of sniffs directed at the familiar and novel mouse was also quantified from video footage.

### Instrumental peak procedure

Mice were placed on a restricted food diet of approximately 2 g of standard chow each day. The chow (Purina, St. Louis, MO, USA) was given in their home cage after behavioral procedures were complete. Animals were weighed daily, and their body weights were maintained at 85−90% of their non-restricted body weight. Mice were trained in 8 standard mouse operant chambers (Med Associates, Fairfax, VT, United States) to lever press for a chocolate pellet (Bio-serv, Frenchtown, NJ, USA). In the first and second training sessions, each response the mouse made immediately delivered a single 20-mg food pellet into a food magazine. Mice were then trained on a fixed interval (FI) 15 s schedule for 7 sessions followed by a FI 25 s schedule for 7 sessions. Under these schedules, a response delivered a pellet only after an elapsed interval from the previous reward delivery. Responses before the interval elapsing produced no pellets. The session terminated after mice earned 20 pellets or 20 min elapsed.

### Instrumental reversal learning

A separate set of mice was tested under instrumental reversal learning. Mice were placed on a restricted food diet of approximately 2 g of standard chow each day. The chow was given in their home cage after behavioral procedures were complete. Animals were weighed daily, and their body weights were maintained at 85−90% of their non-restricted body weight. While placed on food restriction, mice were simultaneously habituated to the operant conditioning chamber for one 15-min session. The next day, mice were conditioned to find pellets in the food cup in a 20-min session in which food pellets were dispensed on a random-time 60-s schedule. Levers were retracted during this phase. The next day mice were trained to use levers. During each session, a single lever was inserted into the operant conditioning chamber. Each lever press resulted in the delivery of a single 20-mg grain-based chocolate-flavored food pellet into the food cup. The session terminated after either 20 lever presses or 20 min elapsed from the start of the session. The mice completed two training sessions per day, one session with the left lever and one session with the right lever.

After acquiring a lever press response mice were placed on a reversal-learning task as previously described (Eisenberg et al., 2021). In this phase, both levers were extended simultaneously. One lever was designated as the rewarded lever and the other lever as the non-rewarded lever. For each trial, responses on the rewarded lever always produced a food pellet, and responses on the non-rewarded lever never delivered a pellet. When no pellet was attained, both levers were retracted for 3 s and reinserted. After the mouse earned between 10 and 14 food pellets, the contingencies assigned to each lever were reversed; responses on the previously rewarded lever produced no pellets, whereas responses on the non-rewarded lever produced pellets. Mice remained in the operant chamber until 80 trials were completed/day or 1 h had passed since the start of the session. Mice underwent 13 reversal-learning sessions, one session per day. The training and testing regimen was completed in approximately 5 weeks, with 1 week of food restriction and habituation to the chambers, 1 week of instrumental training, and 3 weeks of reversal learning.

### Immunohistochemistry

Animals were deeply anesthetized with ketamine/xylazine and perfused with 4% paraformaldehyde (PFA) in phosphate buffered saline (PBS). Brains were removed and post-fixed in 4% PFA/PBS overnight at 4°C, then cryopreserved with 30% sucrose. Sections (12 μm) were cut using a Leica cryostat and mounted onto charged slides. Sections were permeabilized with 0.5% triton in PBS for 20 min and blocked with 1% bovine serum albumin (BSA) and 5% donkey serum in PBS for 1 h at room temperature (RT). Primary and secondary antibodies were prepared in 1% BSA. Sections were incubated with primary antibodies overnight at 4°C in a humidified chamber. Sections were then washed 3 times with PBS for 15 min each. All secondary antibodies were diluted at 1:1000 and incubated for 1 h at RT and washed three times with PBS for 15 min each. Sections were mounted using 4’,6-diamidino-2-phenylindole (DAPI) Fluoromount-G (Southern Biotech #0100−20). Controls for immunostaining included incubation with secondary antibodies in the absence of primary antibodies. Antibodies used: goat anti-p75 (R&D AF367, RRID:AB_2152638, 1:500), rabbit anti-Calbindin (ab49899 RRID:AB_1267903, 1:1000), donkey anti-goat Alexa Fluor 488 (Fisher Scientific #A-11055), donkey anti-goat Alexa Fluor 555 (Fisher Scientific # A-21432), donkey anti-rabbit Alexa Fluor 647 (Fisher Scientific # A-31573).

For the Td-Tomato detection, no antibody was used, since the fluorescence intensity of the marker was strong enough to be detected without immunostaining. Horizontal sections (12 μm) were cut using a Leica cryostat and mounted onto charged slides. Sections were mounted using DAPI Fluoromount-G (Southern Biotech #0100−20). Pictures of the entire cerebellum were taken using a Zeiss LSM 510 Meta confocal microscope, objectives EC Plan-Neo 10X/0.30 M27 and Plan Apo 20X/0.8. Filter configuration for imaging: For the blue channel we used a 405 excitation laser with an HFT 405/488/543/633, emission was detected using BP 420−480. For the green channel, we used a 488 excitation laser with an HFT 405/488/543/633, emission was detected using LP 560. For the red channel, we used a 543 excitation laser with an HFT 405/488/543/633, emission was detected using LP 560. For the far red channel, we used a 633 excitation laser with an HFT 405/488/543/633, emission was detected using LP 560.

### Statistical analysis

For behavioral experiments involving a single measurement (e.g., distance traveled in the open field) we compared genotypes using independent samples *t*-tests. For behavioral experiments with multiple within-subject measurements (e.g., sniffs directed at different objects), we used 2-Way ANOVA’s with within and between-subject factors. If no interaction was detected between the two factors (e.g., genotype and novel/familiar), we proceeded to analyze the single factor (e.g., novel/familiar) within the results of the two-way ANOVA. When a significant interaction was detected between the factors, we proceeded with a simple effect analysis. Within-subject planned comparisons were made using paired *t*-tests, and comparisons between genotypes were made using independent samples *t*-tests with Bonferroni correction.

To correlate recombination levels with a behavioral response, we calculated a recombination index. Using FIJI (Image J), the area of the IGL was measured based on Dapi staining. An ROI was obtained from the Dapi signal and established as the total area. Using this ROI as a mask, we calculated the area positive for Td-Tomato (recombined area). The index was obtained by dividing the recombined area over the total area. Only the cerebellum from animals injected with tamoxifen was used to calculate the correlation index, since no expression of TD-Tomato was detected in the animals without tamoxifen. The recombination index was regressed against different behavioral measurements (e.g., time spent in closed arms of the zero maze), and R-squared was calculated along with estimates of goodness-of-fit.

## Results

### Deletion of *p75NTR* from granule cell precursors

*P75NTR* is widely expressed during embryonic development; its expression starts as early as the blastocyst stage (Vandamme and Berx, 2019). In the nervous system, *p75NTR* is expressed in multiple populations in the developing brain including the cerebellum and the hippocampus. During cerebellar development, p75NTR is expressed in the proliferating GCPs and is downregulated upon cell cycle exit. P75NTR is also expressed in postmitotic Purkinje cells, suggesting a different role for the receptor in this cell population. In adult animals, however, p75NTR expression is restricted to a small number of brain areas, such as the basal forebrain (Lee et al., 1998) and the Purkinje cells in the cerebellum (Carter et al., 2003). To specifically evaluate the role of p75NTR in GCPs and the effects on cerebellar behavior, we used two conditional mouse lines. In both mice lines deletion of p75NTR was under the control of the *Atoh1* promoter, with the difference that in one of the mouse lines deletion of p75NTR was regulated by the normal expression of Atoh1 [p75Atoh1-Cre] while the other mouse line, *Atoh1* expression was tamoxifen-inducible [p75Atoh1-CreERTM] (see methods), which allows better temporal regulation of p75NTR deletion. Additionally, the p75Atoh1-CreERTM mice also have a Td-Tomato reporter, to track the cells that underwent recombination.

**FIGURE 1:**
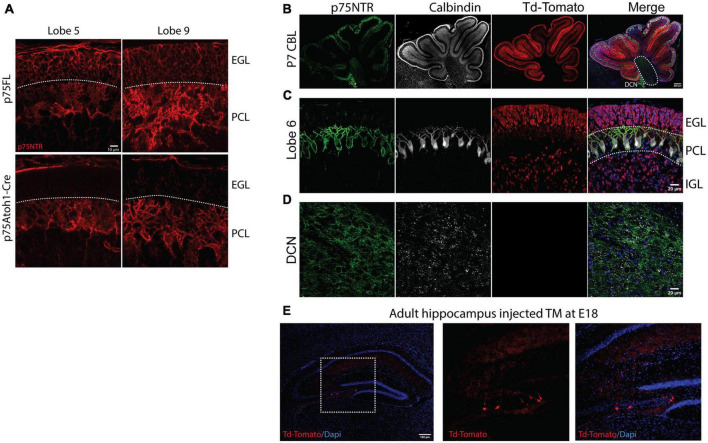
Loss of cerebellar p75NTR. Immunostaining of cerebellar sections. **(A)** Immunostaining for p75NTR in control (p75FL) and p75Atoh1-Cre P7 mouse pups. Note the deletion of p75NTR specifically from the GCPs located in the external granule layer in two different cerebellar lobes. **(B–D)** Immunostaining for p75NTR (green), Calbindin, a Purkinje cell marker (white), and Td-Tomato (red) for p75Atoh1-CreERTM P7 mouse pups injected with tamoxifen. Note the expression of the reporter Td-Tomato only in the GCPs in the EGL and the differentiated granule cells in the internal granule layer **(C)**, no expression of the reporter was observed in the deep cerebellar nuclei **(D)**. **(E)** Expression of Td-Tomato in the hippocampus. With the late delivery of tamoxifen (E17/18), only a reduced number of cells were labeled with tamoxifen. (EGL) external granule layer, (PCL) Purkinje cell layer, (IGL) internal granule layer.

The p75Atoh1-Cre mice have a deletion of p75NTR from GCPs in all lobes in the cerebellum but maintain wildtype expression in Purkinje cells (Figure 1A). In the cerebellum, *Atoh-1* is expressed in all the rhombic lip derivatives, which include the GCPs and the excitatory neurons of the cerebellar nuclei (MacHold and Fishell, 2005; Wang et al., 2005). Therefore, to target more specifically the GCPs in the cerebellum, we took advantage of the late development of this cell population in comparison with the rest of the rhombic lip derivatives. We exposed the mice to tamoxifen by oral gavage of pregnant females at E17/18. With this tamoxifen exposure paradigm, we specifically targeted the deletion of p75NTR in the cerebellar granule cells, indicated by the robust expression of Td-Tomato (Figures 1B, C), without affecting the cerebellar nuclei (Figure 1D). We used these two mouse models to evaluate the role of the cerebellar granule cells in cerebellar function. *Atoh1* is also expressed in the hippocampus; however, injecting tamoxifen at the late stages of embryonic development (E17/18) induced the recombination of only a small number of neurons, leaving the great majority of cells unaffected (Figure 1E).

### Social behavior deficits induced by loss of cerebellar CGN *p75NTR*

**FIGURE 2:**
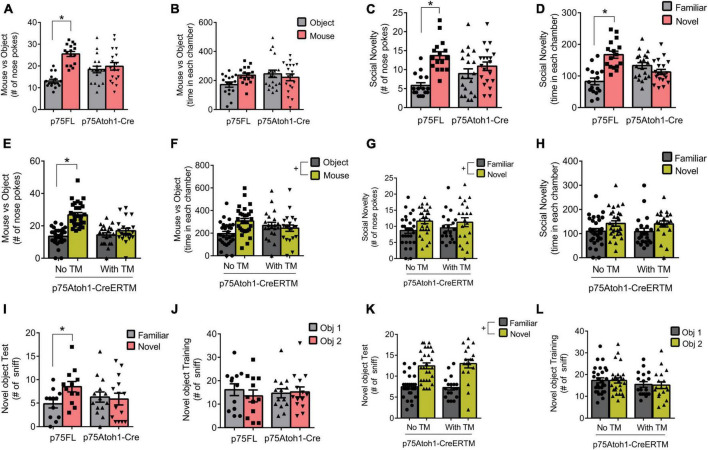
Loss of p75NTR alters social behavior. **(A)** p75Atoh1-Cre mice show no difference in nose pokes directed at a mouse versus non-social object, unlike control mice which show significantly more mouse-directed nose pokes. **(B)** This genotype effect was not observed when examining time spent in the chambers housing the mouse or object. **(C,D)** p75Atoh1-Cre mice show no difference in nose pokes **(C)** and time in chamber **(D)** between novel and familiar mice, unlike control mice which show significantly more nose pokes and chamber time directed at the novel mouse. **(E)** p75Atoh1-CreERTM with TM show no difference in nose pokes directed at a mouse versus non-social object, unlike control mice. **(F)** This treatment effect was not observed when examining time spent in the different chambers. No effect of TM was observed on nose pokes **(G)** and time spent in chamber **(H)** between novel and familiar mice. **(I–L)** Novel object recognition test. **(I)** p75Atoh1-Cre mice show no difference in nose pokes directed at a novel versus familiar object, unlike control mice, which show a significant preference for the novel object. **(J)** This effect was not due to biased object exploration in the training phase. **(K,L)** No effect of TM was observed in novel object exploration in p75Atoh1-CreERTM mice. All data are expressed as the mean ± S.E.M. *Significantly different at *p* < 0.05. ^+^Significant main effect of object or novelty factor.

We evaluated social behavior in the p75Atoh1-Cre and p75Atoh1-CreERTM mice using tests of sociality and social novelty (Figures 2A–H). In the sociality test, we presented mice with a novel mouse in one chamber and an inanimate object in the other, and quantified the number of nose pokes directed at the mouse or object as well as time spent in each chamber. Analysis of nose pokes revealed that loss of p75NTR significantly altered performance in the sociality test. p75Atoh1-Cre mice showed no preference for the mouse over the object, unlike control mice who directed significantly more nose pokes at the mouse compared to the object [ANOVA interaction *F*(1,32) = 20.19, *p* = 0.0001; paired *t*-test object versus mouse: p75FL *p* = 0.0001; p75Atoh1-Cre *p* = 0.4295] (Figure 2A). We observed similar results when analyzing the tamoxifen (TM) inducible mice (Figure 2E). Adult p75Atoh1-CreERTM mice with tamoxifen exposure during embryonic stage E17/18 showed no preference for the mouse over the object, unlike control mice [ANOVA interaction *F*(1,48) = 21.99 *p* = 0.0001; paired *t*-test object vs. mouse: no TM *p* = 0.0001; with TM *p* = 0.0924] (Figure 2E). In contrast to the nose-poke results, time spent in the chamber containing the mouse or object showed no significant interaction with genotype for p75Atoh1-Cre (*p* = 0.0854) or for p75Atoh1-CreERTM mice (*p* = 0.5954) (Figure 2B). p75Atoh1-CreERTM mice with or without TM spent significantly more time with the mouse over the object (ANOVA main effect of object/mouse factor *F*(1,48) = 7.851, *p* < 0.0073) (Figure 2F). Overall, the results from nose-poke behavior suggest that loss of p75NTR from cerebellar GCN’s alters preference for social interactions.

During the social novelty test, we confined a novel mouse to one chamber of the arena, and evaluated nose pokes and the time spent with either the novel or the familiar mouse. Among p75Atoh1-Cre mice, analysis of nose pokes revealed that loss of p75NTR significantly modulated preference for social novelty. p75Atoh1-Cre mice showed a modest preference for the novel mouse compared to the familiar mouse, unlike control mice, which directed significantly more nose pokes at the novel mouse [ANOVA: interaction *F*(1,34) = 15.10, *p* = 0.0004: paired *t*-test familiar vs. novel: p75FL *p* = 0.0001; p75Atoh1-Cre (*p* = 0.0573) (Figure 2C)]. Similarly, p75FL mice spent significantly more time in the chamber housing the novel mouse, whereas p75Atoh1-Cre mice spent a similar amount of time in each chamber [ANOVA: interaction *F*(1,34) = 10.96, *p* = 0.0022; paired *t*-test familiar vs. novel: p75FL *p* = 0.0011; p75Atoh1-Cre *p* = 0.2202] (Figure 2D).

In contrast, we found no significant effects of genotype on social novelty among p75Atoh1-CreERTM mice. We found no significant interaction involving genotype and novelty for nose pokes or chamber time (*p’s* > 0.6) (Figure 2G). We observed a main effect of novelty on nose-pokes [*F*(1,48) = 4.101 *p* = 0.0484] and on chamber time that approached significance [*F*(1,48) = 3.9 *p* = 0.0541] (Figure 2H), suggesting that, independent of genotype, mice showed a preference for novel social interactions.

Taken together, the results from the sociality test suggest that loss of p75NTR, either in constitutive or inducible-Cre mice, reduces social-directed nose-poke behavior in the sociality test. Constitutive loss of p75NTR also reduces novelty-directed investigatory behavior in the social novelty test, although this effect was not observed in the inducible-Cre mice.

### Novel object recognition

Object identity memory was assessed in the novel object recognition test. We found that constitutive loss of p75NTR disrupted performance in the task. p75Atoh1-Cre mice showed no preference for the novel object, while control mice preferred to explore the novel object over the familiar one [ANOVA: genotype x object interaction *F*(1,24) = 23.20; *p* = 0.0001]. In the single factor analysis, control p75FL mice nose-poked the novel object significantly more frequently (*p* = 0.0004); p75Atoh1-Cre, in contrast, showed no significant difference in nose-pokes toward the novel and familiar objects (*p* = 0.3962) (Figure 2J). Interestingly, we found no genotype effect on social novelty in p75Atoh1-CreERTM mice. Mice with or without TM showed a significant preference to explore the novel object over the familiar object. No significant genotype x object interaction was observed (*p* = 0.5623). A significant main effect of novelty was observed [*F*(1,44) = 75.14 *p* = 0.0001], indicating that these mice investigated the novel over familiar object independent of genotype (Figure 2L). During the training session, there was no biased exploratory preference for either of the two identical objects in p75Atoh1-Cre (*p* = 0.2276) (Figure 2I) or p75Atoh1-CreERTM mice (*p* = 0.7253) (Figure 2K).

These results indicate that p75Atoh1-Cre mice were impaired in the novel object recognition task. The lack of effect in p75Atoh1-CreERTM might be due to the defects induced by the absence of p75NTR in other areas outside the cerebellum, perhaps the hippocampus.

### Increased anxiety in p75Atoh1-Cre but not p75Atoh1-CreERTM mice

**FIGURE 3:**
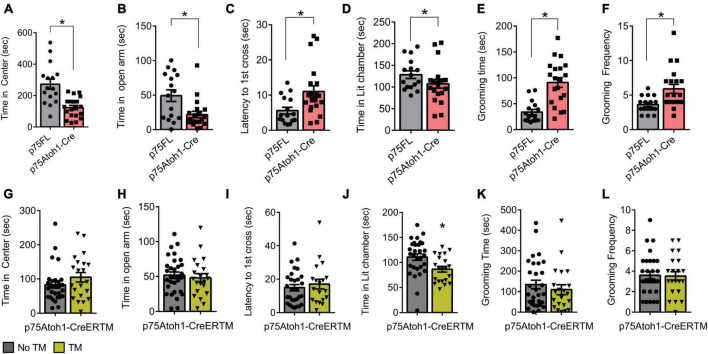
p75Atoh1-Cre mice show increased anxiety-related behavior. **(A–F)** p75Atoh1-Cre show differences in: **(A)** time spent in the center of the open-field arena; **(B)** time spent in the open arms of the elevated zero maze; **(C)** latency to cross to the illuminated chamber in the light-dark transition test and **(D)** time spent in the illuminated chamber; **(E)** time self-grooming, and **(F)** frequency of grooming bouts. In contrast p75Atoh1-CreERTM mice treated with TM did not differ from controls on any measure **(G–L)**. *p < 0.05 Student *t*-test; mean ± S.E.M.

We observed anxiety-related behavior in p75Atoh1-Cre but not p75Atoh1-CreERTM mice in multiple tests. In the open field test, p75Atoh1-Cre mice spent significantly less time in the center of the arena compared to controls (*p* = 0.0001) (Figure 3A). Similarly, in the elevated zero maze, p75Atoh1-Cre mice spent significantly less time in the zero maze’s open arms compared to control mice (*p* = 0.0048) (Figure 3B). Likewise, in the light-dark transition test, p75Atoh1-Cre mice were slower to enter the lit chamber compared to control mice (*p* = 0.0105) (Figure 3C), although we did not observe any significant difference in the time spent exploring the lit chamber between the two groups (*p* = 0.136) (Figure 3D). We additionally observed that p75Atoh1-Cre engaged in longer self-grooming bouts compared to control mice (*p* = 0.0001) (Figure 3E), and more frequent grooming (*p* = 0.0059) (Figure 3F). Taken together, the performance of p75Atoh1-Cre mice on these tests is consistent with an elevated anxiety phenotype.

In contrast, p75Atoh1-CreERTM mice with or without tamoxifen showed no differences in anxiety behavior. There was no significant difference in the cumulative time spent in the center of the arena between groups (*p* = 0.1679) (Figure 3G). Similarly, in the elevated zero maze, both groups of mice spent similar time in the zero maze’s open arms (*p* = 0.6208) (Figure 3H). In the light-dark transition test, there was no difference in the latency to first cross into the illuminated chamber between the two groups (*p* = 0.2513) (Figure 3I); however, p75Atoh1-CreERTM spent less time exploring the lit chamber compared to controls (*p* = 0.0108) (Figure 3J). In the grooming test, no difference was observed in the total time of self-grooming bouts between the two groups longer self-grooming bouts compared to control mice (*p* = 0.4504) (Figure 3K), or the frequency of grooming longer self-grooming bouts compared to control mice (*p* = 0.8931) (Figure 3L). Taken together, these data suggest that loss of p75NTR specifically in early-developing granule cells of the cerebellum may not produce the anxiety phenotype seen with the p75Atoh1-Cre mice.

### Loss of p75NTR does not affect basic locomotor parameters

Loss of p75NTR did not affect basic locomotor parameters in the open field. P75Atoh1-Cre were no different from controls in velocity (*p* = 0.7331) and total distance traveled (*p* = 0.9465). Similarly, we observed no difference between p75Atoh-CreERTM and controls in velocity (*p* = 0.6331) and distance traveled in the open field test (*p* = 0.4432) (Supplementary Figure 1). Therefore, the effects observed in the different behavioral tests are not due to an inability to move about the arena.

### The recombination induced by tamoxifen was highly variable

**FIGURE 4:**
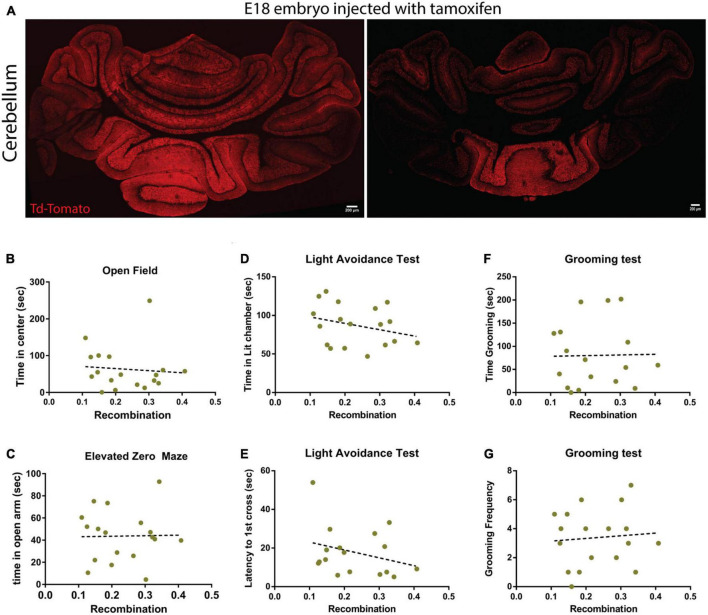
Recombination levels do not correlate with behavior in p75Atoh1-CreERTM mice. Analysis of the recombination level of p75Atoh1-CreERTM adult mice after tamoxifen injection during development. **(A,B)** Immunostaining for Td-Tomato (red) and Dapi (blue) in p75Atoh1-CreERTM adult mouse injected with tamoxifen at embryonic day 17/18. **(A)** Expression of Td-Tomato in the cerebellum of two sibling mice that received the same tamoxifen doses. Note the high variability in the recombination levels. **(B–G)** Correlation analysis between the level of recombination and the behavioral results shown in this figure. The brains of all the mice injected with tamoxifen used in this figure were obtained, sectioned, and stained for Td-Tomato as a proxy of recombination level. **(B)** Correlation between the level of recombination and the time spent in the center of the open field arena. **(C)** Correlation between the level of recombination and the time spent in the open arm of the elevated zero maze. **(D,E)** Light-dark transition test: correlation between the level of recombination and **(E)** the time spent in the lit chamber or **(D)** the delay time to first cross to the illuminated chamber. **(F,G)** Correlation with the grooming test: correlation between the level of recombination and **(F)** the time the mice spent grooming or **(G)** the frequency of grooming.

The p75Atoh1-CreERTM expresses a Td-Tomato reporter allowing assessment of the recombination efficiency following tamoxifen treatment. After finishing the battery of behavioral tests on the mice injected with tamoxifen, we collected the brains and confirmed the recombination efficiency in these animals. Surprisingly, even though the animals received the same dose of tamoxifen (pregnant female received the tamoxifen treatment), we observed a high degree of variability in the level of recombination induced by the exposure to tamoxifen (Figure 4A). To examine the possibility that lower transfection levels may explain the discrepancy between p75Atoh1-Cre and p75Atoh1-CreERTM mice, we correlated behavior with the level of recombination in the cerebellum. If a strong correlation between the recombination level and the behavioral response is observed, this might indicate that the granule cells might be involved in anxiety-like behavior, but in the animals with low recombination efficiency, we may not have reached a threshold of recombined cells to affect the behavior. When comparing behavioral measures against recombination density (see methods), we observed no significant correlation with any of the anxiety-like behavior measures analyzed (open field *R*^2^ = 0.0074; elevated zero maze *R*^2^ = 0.0003; light transition test time in lit chamber *R*^2^ = 0.0815; light transition test latency to 1st cross *R*^2^ = 0.088; grooming test time *R*^2^ = 0.005, grooming test frequency *R*^2^ = 0.007) (Figures 4B–G). These findings confirm that even in the animals with high-recombination level, removing p75NTR from granule cells does not affect anxiety-related behaviors, supporting our previous conclusion that loss of p75NTR expression in granule cells progenitors, with the developmental consequences for proliferation and circuit development previously described (Zanin et al., 2016, 2019), may not produce an anxiety phenotype.

### Loss of *p75NTR* using the Atoh1 promoter has no observable effect on reward learning

**FIGURE 5:**
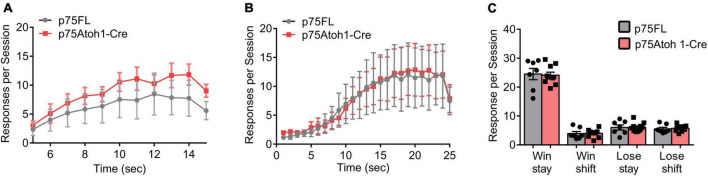
Loss of p75NTR spares performance in the reward-based operant tasks. **(A,B)** Responses during the fixed instrumental peak procedure. The trial interval was divided into 1-s bins, and lever presses were binned according to the time point they occurred during the trial interval. Responses per session were averaged for each mouse, and the group means, and SEM is illustrated in the plot for mice trained under an FI-15 schedule **(A)** and FI-25 schedule **(B)**. **(C)** Responses during the reversal learning task. Responses were classified as win stay, win shift, lose stay or lose shift, depending on the current trial’s response and the preceding trial’s outcome and response (see methods for additional description). Responses per session were averaged for each mouse, and the group means, and SEM is illustrated in the plot in panel **(C)**.

We examined the behavior of p75Atoh1-Cre mice in two reward-learning tasks that have recently been linked to cerebellar function. In the instrumental peak procedure mice learned over multiple sessions to time their response toward the end of the fixed interval. We trained mice on two intervals, an FI-15 s interval, followed by an FI-25 s interval. Mice showed significantly greater allocation of responses near the end of the interval compared to the beginning (Figures 5A, B). Responses were significantly greater 15- or 25-s after the trial start compared to 5 s after the trial start (ANOVA: main effect of trial time *p’s* < 0.001). We found no effect of genotype or interaction for either interval (*p*’s > 0.9), indicating that all mice learned to time their responses to the interval schedule and that loss of p75NTR in cerebellar GCPs did not influence learning in this task.

In the reversal learning task, mice chose between two levers- one lever delivered a reward and the opposite lever had no effect. The lever-reward contingencies were reversed every 10−14 rewarded trials. Mice had to adapt their responses to the changing reward contingencies. We classified responses in each trial into one of four categories, depending on the choice and outcome of the previous trial: win-stay responses occurred when animals repeated a previously rewarded action; win-shift responses occurred when animals shifted to a new action following a reward; lose-stay responses occurred when animals repeated an action following no reward; lose-shift responses occurred when animals shifted actions following no reward. As shown in Figure 5C, mice predominately engaged in win-stay responses. Following loss trials, mice engaged in equal numbers of lose-shift and lose-stay responses. Loss of p75NTR did not affect behavior in this task. We found no effect of genotype on the allocation of responses in the task (*p’s* > 0.8), indicating that loss of p75NTR in GCPs does not impact behavioral flexibility in a reversal-learning task.

## Discussion

Our previous studies have demonstrated that the absence of p75NTR from developing GCPs alters their cell cycle regulation, resulting in excess production of CGNs. The excess number of CGNs, in turn, alters the excitatory-inhibitory balance of inputs to Purkinje cells. In our present work, we find that mice with an excess number of CGNs show deficits in tests of sociality. These results suggest that perturbations of CGN development by loss of p75NTR disrupt social engagement in mice, which has implications for understanding the etiology of neurodevelopmental disorders that feature social behavior deficits, such as Autism Spectrum Disorder.

The granule cells receive a large variety of signals from the mossy fibers; this includes sensory, motor, and contextual information. Sensory-motor stimulus (joint angle, visual and whisker cues, head position, body velocity, etc.) is encoded in a group of CGN, allowing the system to combine multiple stimuli to generate an accurate representation of the behavioral context necessary to control behavior. The granule cells will re-code this information in a way that can be recognized and learned by their targets, the Purkinje cells. Hence, a quantitative imbalance between the different neuronal populations of the cerebellum (mossy fibers, CGN, and PC) is likely to affect the encoding of information leading to suboptimal responses. For instance, one of the most consistent findings in postmortem analysis of autistic patients is a loss of Purkinje cells (Kern, 2003; Skefos et al., 2014), with no apparent loss of the other neuronal types. Therefore, the disabilities observed in these patients might be explained, at least in part, by the disruption of the information flow caused by the quantitative imbalance between the CGN and PC populations. An excess of granule cells, such as the one observed in the p75Atoh1-Cre mice could potentially “dilute” the information received from the mossy fibers. In our previous publications, we have confirmed that Purkinje cells in these mice have an increased firing frequency (Zanin et al., 2019), consistent with an excess of excitatory input, likely from the excess CGN. Although we did not measure the activity in the deep cerebellar nuclei (DCN), the alteration in PC activity most likely will compromise the output information of the DCN to the rest of the brain. Moreover, substantial evidence suggests that learning occurs in the Purkinje cells, however, new experimental data predicts that learning would also take place at the cerebellar input stage — the granular layer (Giovannucci et al., 2017). Therefore, an excess number of CGNs can compromise the contextual information transfer to the PC, as well as the information processing at the CGN level.

### How does the absence of *p75NTR* induce social behavior deficits?

Several studies have established the role of the cerebellum in the social domain, including emotion and intentions (Schmahmann and Sherman, 1998; Baumann et al., 2015; Guell et al., 2018). Thus, it has been hypothesized that the cerebellum may modulate non-motor behavior, such as social behavior, in the same way it modulates motor control (Andreasen and Pierson, 2008; Stoodley and Schmahmann, 2010). Recently it was shown that the cerebellum can carry information about reward expectation (Wagner et al., 2017; Carta et al., 2019), and its known that crus I projects to somatosensory and anterior cingulate cortex, areas highly involved in social interactions, therefore encoding information that is necessary for the expression of some forms of behavior. The excess of CGN observed in the *p75P75Atoh1-Cre* could generate a suboptimal context representation in these mice, affecting their social interaction responses. Previously, we demonstrated that mice lacking p75NTR expression in the cerebellar GCP (p75Atoh1-Cre) have motor (Zanin et al., 2016) and associative learning deficits (Zanin et al., 2019). In the present work, we extended our findings and demonstrated that these mice also present social interaction deficits. In the mouse vs. object test, both sets of conditional mice (p75Atoh1-Cre and p75Atoh1-CreERTM with tamoxifen) made similar numbers of investigatory nose pokes toward the object and the mouse, in contrast to control mice (p75FL or p75Atoh1-CreERTM without tamoxifen, respectively), which prefer the mouse over the object. In the social novelty test, p75Atoh1-Cre showed no preference for the familiar over the novel mouse, in contrast to control mice, which show a significant preference for the novel mouse, represented as the number of sniffs toward the novel mouse. In contrast, we found no effects on social novelty of p75NTR deletion in the TM-inducible Cre mice. This may indicate that social novelty preference, as opposed to choices between social and non-social encounters, is less sensitive to p75NTR deletion. Although we observed some Td-Tomato recombination in the hippocampus of p75Atoh1-CreERTM injected with tamoxifen, the actual number appears extremely low to have had an impact on social behavior, further supporting our hypothesis that sociality (mouse versus object) deficits in these animals is driven by the excess of CGN generated during development. Taken together, the results obtained from both mouse lines strongly suggest that removing p75NTR from GCP during development is sufficient to induce sociality alterations in adult animals. These findings highlight the role of p75NTR as a potential susceptibility gene in neurological disorders with ontogeny in developmental deficits.

Interestingly, we observed some differences between the conditional (p75Atoh1-Cre) and the tamoxifen-inducible mice (p75Atoh1-CreERTM). In the anxiety-related behaviors, e.g., open field, grooming, elevated zero maze, and light transition test, p75Atoh1-Cre mice performed significantly different than control mice (p75FL) suggesting elevated anxiety in these animals. However, no difference was observed between the p75Atoh1-CreERTM with and without tamoxifen. One possible explanation for these differences is that another neuronal population that expresses Atoh1 and p75NTR is responsible for the behavioral difference only observed in the p75Atoh1-Cre. In mice, Atoh1 is first detected at E9 and continues throughout development, but it is absent from adult brains, although, the specific location of Atoh1 expression is controversial. Using *in situ* hybridization and immunostaining, Atoh1 expression was detected in the cranial ganglia, the dorsal wall of the neural tube, and the hindbrain, there is also a strong expression of Atoh1 in the rhombic lip, which contains the GCPs of the cerebellum as well as the excitatory cells of the deep cerebellar nuclei (Akazawa et al., 1995; Ben-Arie et al., 1996, 1997). Using a Cre reporter line, besides the above-mentioned places, Atoh1 was also found in the dentate gyrus in the hippocampus (Lumpkin et al., 2003); consistently, we identified a small number of Td-Tomato positive cells in the p75Atoh1-CreERTM injected with tamoxifen, suggesting that Atoh1 is present during hippocampal development. P75NTR is expressed in the developing hippocampus (Koh et al., 1989; Zuccaro et al., 2014). The hippocampus and the cerebellum are both involved in anxiety behaviors (Bannerman et al., 2004; McHugh et al., 2004; Engin and Treit, 2007; Phillips, 2015; Moreno-Rius, 2018), therefore the elevated anxiety observed only in the conditional p75Atoh1-Cre might be due to developmental defects in the hippocampus induced by the deletion of p75NTR after Atoh1 is expressed. This is supported by the findings of a small proportion of recombined neurons in the p75Atoh1-CreERTM mice after tamoxifen injection. The late tamoxifen injection seems to impact a reduced number of cells, therefore the great majority of cells in the hippocampus would still maintain wild-type expression of p75NTR; thus, no impact on anxiety levels (as well as performance on the novel object recognition test) was observed in these animals.

An alternative explanation is that the p75Atoh1-CreERTM might not reach the threshold of excess CGN to affect anxiety-related behavior. The absence of p75NTR accelerates the GCP cell cycle, generating an excess of granule cell neurons (Zanin et al., 2016, 2019). In the p75Atoh1-Cre mouse, the entire GCP population lacks p75NTR expression, and a full penetrance of recombination was expected, generating the highest excess of CGN number; however, even in the highest level of recombination in the p75Atoh1-CreERTM we did not observe a 100% penetrance, suggesting that only a fraction of the GCP lack p75NTR, and thus fewer excess CGN are generated in these animals. Further experiments that allowed more broad deletion of the expression of p75NTR specifically from the GCP are required to completely rule out whether an excess of granule cells would also affect anxiety-related behaviors. It is worth mentioning that both mice models used in our study affect CGN development; however, our findings do not directly address how an excess number of neurons alter the activity of the CGN. Further studies that evaluate neuronal activity are required to answer these questions.

The extensive range of behaviors affected by the absence of p75NTR might be explained by the expression pattern of this receptor and the multiple behaviors in which the cerebellum is involved. P75NTR is expressed in every proliferating GCP. In our previous work, we demonstrated that the absence of the receptor induced defects in proliferation and migration, and these cellular defects were observed in both the anterior and posterior lobes in the cerebellum. Each area of the cerebellum is involved in different aspects of cerebellar function; therefore, it is possible that the defects induced by p75NTR will affect every folium of the cerebellum impacting multiple behaviors in adult animals. Further behavioral tests would likely elucidate other behaviors impacted by the excess of CGN. Regardless, our research identifies p75NTR as a potential risk factor in cerebellar development that contributes to the growing evidence by which the cerebellum impacts non-motor as well as motor behaviors.

## Data availability statement

The datasets presented in this study can be found in online repositories. The names of the repository/repositories and accession number(s) can be found below: https://figshare.com/articles/dataset/Excess_cerebellar_granule_neurons_induced_by_the_absence_of_p75NTR_during_development_elicit_social_behavior_deficits_in_mice/21919035.

## Ethics statement

This animal study was reviewed and approved by Rutgers IACUC.

## Author contributions

JZ, WF, and MS conceived the experiments and wrote the manuscript. JZ, MP, and DE performed the experiments. All authors contributed to the article and approved the submitted version.
