# Supplementary figures and images for: Excess cerebellar granule neurons induced by the absence of p75NTR during development elicit social behavior deficits in mice
Source: Front Mol Neurosci. 2023 May 25;16:1147597. doi: 10.3389/fnmol.2023.1147597 (PMC10249730; doi:10.3389/fnmol.2023.1147597)

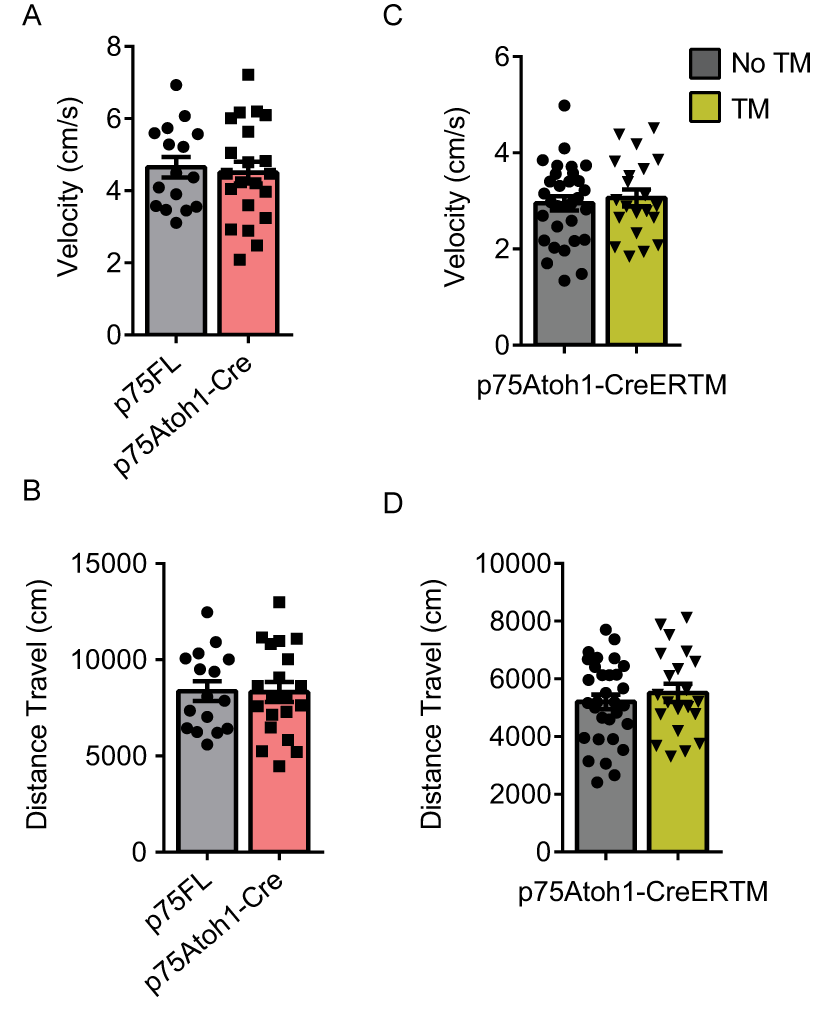

Supplement: Supplementary file 1 [file Image_1.TIF]
